# Activity of Ceftaroline against Aerobic Gram-Positive and Gram-Negative Pathogens: Effect of Test Method Variability

**DOI:** 10.5402/2011/787290

**Published:** 2011-11-17

**Authors:** Diane M. Citron, Yumi A. Warren, Kerin L. Tyrrell, Ellie J. C. Goldstein

**Affiliations:** ^1^R.M. Alden Research Laboratory, Culver City, CA 90230, USA; ^2^Department of Medicine, David Geffen School of Medicine, Los Angeles, CA 90095, USA

## Abstract

Ceftaroline is a new cephalosporin with bactericidal activity against methicillin-resistant *S. aureus* (MRSA) as well as gram-negative pathogens. Variations of in vitro test conditions were found to affect ceftaroline activity, with 5% NaCl inhibiting growth and/or reducing the minimum inhibitory concentrations (MICs) for *E. coli, K. pneumoniae, M. catarrhalis, H. influenzae,* and streptococci, while an inoculum of 10^6^ CFU/mL raised MICs of some *E. coli, K. pneumoniae,* and M. catarrhalis strains.

## 1. Introduction

The emergence of MRSA has spurred the development of alternative therapies such as daptomycin, linezolid, and quinupristin-dalfopristin, which are not active against gram-negative pathogens and require combination therapy. Ceftaroline is a new, parenteral, broad-spectrum cephalosporin with bactericidal activity against MRSA, including vancomycin-intermediate (VISA) strains, and multidrug-resistant *Streptococcus pneumoniae* (MDRSP); it is also active against common gram-negative pathogens and can therefore be used as monotherapy for mixed infections [1–6]. Since alterations of in vitro test conditions can potentially affect susceptibility results, we evaluated the effects of 15 variations to the standard test conditions as specified by the Clinical and Laboratory Standards Institute (CLSI) guidelines [7, 8] on the minimum inhibitory concentrations (MICs) of ceftaroline against 30 isolates representing 10 species of clinically important, commonly encountered organisms. 

## 2. Materials and Methods

### 2.1. Standard Method

The CLSI reference broth microdilution method (CLSI 2006, 2009) uses cation-adjusted Mueller Hinton broth (CAMHB) (Difco, BD; Sparks, Md, USA), which has a calcium concentration of 25 mg/L, a magnesium concentration of 12.5 mg/L, and a pH of 7.3 + 0.1. The standard inoculum is 5 × 10^5^ colony-forming units (CFUs)/mL for broth microdilution testing and 10^4^ CFU/spot for agar dilution tests.

### 2.2. Test Variables

Modifications of standard test conditions included adjusting the Ca^++^ content of CAMHB to 50 mg/L Ca, addition of NaCl to 5%, adjusting the broth to pH 6 and pH 8, and using inocula of 10^4^ and 10^6^ colony-forming units (CFUs)/mL. Other variations to the standard medium were the addition of 10% and 50% pooled human serum (Sigma; St. Louis, Mo, USA), the addition of lysed horse blood to 2.5% (LHB) (Hardy Diagnostics, Inc. Santa Maria, Calif, USA), and using *Haemophilus* test medium (HTM) broth. While MIC panels were incubated at 35°C in ambient conditions, for comparative purposes, additional tests in CAMHB were incubated in the anaerobic chamber or in 5% CO_2_.

### 2.3. MIC Test Panel Preparation

Ninety-six-well panels were prepared with twice the final concentration of ceftaroline (50 *μ*L/well) using the Quick-Spense IIe apparatus (Sandy Springs Instruments; Germantown, Md, USA) and stored at −70°C until used. Addition of 50 *μ*L of the organism inocula to the wells reduced the final ceftaroline concentration to the desired level of 0.008 to 8 *μ*g/mL. Some of the organisms did not achieve a ceftaroline MIC endpoint, and further dilutions were prepared to 0.001 *μ*g/mL for retesting some of those isolates. 

### 2.4. Agar Dilution Test Media

Agar dilution MICs were determined on unsupplemented Mueller Hinton agar (MHA) (Difco), with 5% LHB, and on HTM with 1.5% agar (HTMA). Serial twofold dilutions of ceftaroline were added to molten agar deeps to prepare the plates for use on the same day. Concentrations of ceftaroline ranged from 0.008 to 8 *μ*g/mL. Drug-free growth control plates were included (CLSI, 2006).

### 2.5. Test Organisms

All 30 strains tested were recent clinical isolates and American Type Culture Collection (ATCC) quality control (QC) strains, which included *Escherichia coli* ATCC 25922, *Staphylococcus aureus* ATCC 29213, *Enterococcus faecalis *ATCC 29212, *Streptococcus pneumoniae *ATCC 49619, and *Haemophilus influenzae *ATCC 49247. Details about the clinical isolates are listed in Table 1. Clinical isolates were selected based on previously demonstrated resistance patterns. The isolates were stored in 20% skim milk at −70°C and were taken from frozen stock and transferred twice on blood or chocolate agar (Hardy Diagnostics Inc.) before testing.

### 2.6. Inoculum Preparation for Microbroth Dilution Tests

Standard inocula were prepared by suspending colonies from overnight cultures in 0.85% saline to equal the turbidity of the 0.5 McFarland standard and diluting it in CAMHB with the various additives at twice their final concentration, which upon addition of 50 *μ*L of inoculum to the test panel were diluted 1 : 2. The 10^4^ and 10^6^ cfu/mL inocula were prepared by diluting the saline suspension either 10-fold more (for 10^4^ cfu/mL) or 10-fold less (for 10^6^ cfu/mL). The trays were inoculated with 50 *μ*L of cell suspension for a final inoculum of ~5 × 10^5^ CFU/mL which was validated by quantitative subculture from the growth control well. Inoculum preparation and all testing were performed in duplicate.

### 2.7. Agar Dilution Testing

For agar dilution tests, the cell suspensions prepared as above were diluted 1 : 10 in CAMHB and applied to the agar plates using a Steers replicator device that delivered a final inoculum of 10^4^ CFU/spot.

### 2.8. MIC Determinations

After overnight incubation, the broth microdilution trays were examined for growth. The MIC was the lowest drug concentration that completely inhibited growth [7]. For agar dilution, the plates were incubated at 35°C overnight. The MIC was the lowest concentration that completely inhibited growth or resulted in a marked reduction of growth as compared with the drug-free control [7].

## 3. Results

We obtained MICs from duplicate tests under the variations shown in Table 2. In cases of discrepancy, the higher value was recorded. The ceftaroline MICs for the QC isolates (tested with the reference microbroth methods according to CLSI guidelines) were all within their acceptable ranges. Effects of variables in testing were noted where 5% NaCl inhibited growth and/or reduced MICs for *E. coli *and *K. pneumoniae* and completely inhibited the growth of *M. catarrhalis, H. influenzae*, and all streptococci. Using an increased inoculum of 10^6^ cfu/mL increased the MIC 5-fold for 1 of 3 *E. coli* strains that was also resistant to ampicillin (MIC > 32 *μ*g/mL) and 1 of 3 *K. pneumoniae *strains that did not appear to have any unusual resistance pattern (ampicillin MIC 32 *μ*g/mL, ceftriaxone 0.25 *μ*g/mL). This *K. pneumoniae* isolate produced the same result when retested. The higher inoculum also increased MICs 3- to 5-fold for *M. catarrhalis*. The addition of blood or serum to the medium enhanced *M. catarrhalis* growth without changing the MICs. Testing on agar, especially HTMA, produced MICs that were 1–3 dilutions higher. All other variables showed minimal effect, and the MICs were generally within one dilution of the reference method.

## 4. Discussion

Standardization of test conditions is an important factor in reliably reporting MICs. Yet variations of conditions may occur, and diverse adjustments may be employed by researchers for unusual testing circumstances worldwide. Jones et al. [9] studied the effects of modifying five parameters (11 variations) of in vitro testing of ceftaroline against 15 selected isolates. They found inocula of 5 × 10^7^ raised the MICs from 8 to >32 and 0.5 to >32 *μ*g/mL on *P. aeruginosa* and *E. cloacae*, respectively. They also noted that pH 5.0 impaired the growth of 9/12 organisms and consequently lowered their MICs and that increased calcium concentration lowered the MICs of *E. faecalis* and *E. faecium*, but that the addition of serum or LHB and variable incubation conditions did not affect ceftaroline MICs. Our study differed from theirs by including a larger number of isolates and adding such variables as 5% NaCl, serum at 10 and 50% concentrations, variations in incubation conditions, and while there was some overlap in species, they did not report on the respiratory pathogens *H. influenza* and *M. catarrhalis*, nor did they study *S. pyogenes* or *K. pneumoniae*. While some of our test variations were in accord with those of Jones et al., they differed in several respects as well. Jones et al. [9] noted that high inocula did not affect *E. coli* MICs but did raise *E. cloacae* and *P. aeruginosa *MICs, but we found a fourfold increased MIC for one of three *E. coli* strains tested as well as a fivefold increase with one of three *K. pneumonia* isolates, suggesting strain variation. Additionally, we found that *M. catarrhalis*, *H. influenza,* and *S. pyogenes* grew poorly with NaCl supplementation and at pH 6.0. An inoculum of 10^6^ CFU/mL also increased the MICs four- to six-fold for all three *M. catarrhalis* strains tested.

## 5. Conclusion

The in vitro antibacterial activity of ceftaroline was adversely affected by 5% NaCl which inhibited growth and/or reduced MICs for *E. coli*, *K. pneumoniae*, *M. catarrhalis*, *H. influenzae, *and streptococci, while an inoculum of 10^6^ CFU/mL raised MICs of some *E. coli*, *K. pneumoniae*, and *M. catarrhalis* strains. The other modifications tested did not adversely affect MIC results. Organisms with special growth requirements can be tested for ceftaroline susceptibility with reasonable assurance that test conditions will not affect the MIC results.

## Figures and Tables

**Table 1:** List of organisms used in the study.

Organism	RMA number	Specimen source	Date isolated	Comments
*E. coli*		ATCC 25922		
	19089	Blood	3/7/2007	Ampicillin = 4 *μ*g/mL
	19090	Primary infection site	3/28/2007	Ampicillin ≥32 *μ*g/mL

*K. pneumoniae*	19091	Primary infection site	3/1/2007	Ampicillin = 32 *μ*g/mL
	19092	Blood	6/6/2007	Ampicillin = 16 *μ*g/mL
	19093	Blood	6/29/2007	Ampicillin = 32 *μ*g/mL

*H. influenzae*		ATCC 49247		
	16081	Respiratory	12/31/2003	*β*-Lactamase-negative
	18520	Respiratory-sinus	12/23/2005	*β*-Lactamase-positive

*M. catarrhalis*	11940	Respiratory-sinus	6/14/2000	
	14032	Respiratory-sinus	5/22/2002	
	18861	Respiratory-sputum	1/31/2007	

*S. aureus*		ATCC 29213		
	18488	Chest infection site	2/11/2005	Methicillin-S
	18401	Blood	8/16/2005	Methicillin-S
	18483	Head abscess	10/15/2005	Methicillin-R
	18504	Primary infection site	11/18/2005	Methicillin-R
	18526	Blood	10/24/2005	Methicillin-R

*E. faecalis*		ATCC 29212		
	18284	Foot infection site	3/24/2005	
	18877	Blood	4/10/2007	

*S. pyogenes*	17018	Diabetic foot infection site	1/22/2003	
	17019	Diabetic foot infection site	10/22/2002	
	19047	Abdominal lesion	10/26/2007	Clindamycin-R

*S. pneumoniae*		ATCC 49619		
	19094	Ear	10/29/2007	Penicillin-S
	19095	Eye	1/9/2007	Penicillin-S
	13345	Nasopharynx	11/14/2001	Penicillin = 8 *μ*g/mL
	13385	Nasopharynx	12/4/2001	Penicillin = 8 *μ*g/mL
	18876	Eye	1/2/2007	Penicillin = 8 *μ*g/mL

RMA: R.M. Alden (culture collection).

ATCC: American Type Culture Collection.

**Table 2:** Ceftaroline MICs (*μ*g/mL) in the cation-adjusted Mueller-Hinton broth with test variables and agar dilution MICs.

		Ceftaroline MICs (*μ*g/mL) in the cation-adjusted Mueller Hinton broth with test variables													Agar dilution		
			Ca^++^	% NaCl	pH		Inoculum cfu/mL		% serum				Incubation		MHA	MHA/ LHB	HTMA
Organism	RMA number	REF	50 mg/L	5	6	8	10^4^	10^6^	10	50	LHB	HTM	CO_2_	Anaerobic			
	ATCC																
*E. coli*	25922	0.06	0.06	≤0.008	0.125	0.06	0.06	0.125	0.06	0.125	0.125	0.125	0.125	0.03	0.06	0.25	0.25
	19089	0.06	0.125	0.015	0.06	0.06	0.03	0.125	0.06	0.06	0.125	0.06	0.125	0.125	0.125	0.25	0.25
	19090	0.03	0.125	≤0.008	0.06	0.03	0.03	2	0.06	0.03	0.125	0.125	0.125	0.125	0.03	0.25	0.25

*K. pneumoniae*	19091	0.25	0.25	0.015	0.5	0.25	0.25	>8	0.25	0.125	0.25	0.5	0.5	0.25	0.125	0.25	0.5
	19092	0.03	0.125	0.015	0.06	0.06	0.03	0.125	0.06	0.06	0.125	0.06	0.125	0.06	0.06	0.125	0.25
	19093	0.06	0.125	0.015	0.06	0.06	0.03	0.25	0.03	0.06	0.125	0.06	0.125	0.06	0.06	0.125	0.25

	ATCC																
*H. influenzae*	49247	0.015	0.015	ng	ng	0.06	ng	0.06	0.06	0.06	0.06	n/t	0.06	0.03	ng	0.06	0.125
	16081	≤0.008	≤0.008	ng	ng	≤0.008	ng	≤0.008	≤0.008	≤0.008	≤0.008	n/t	≤0.008	≤0.008	ng	0.06	0.015
	18520	0.015	0.015	ng	ng	0.03	0.015	0.06	0.06	0.03	0.03	n/t	0.015	0.03	ng	0.015	0.06

*M. catarrhalis*	11940	0.125	0.06	ng	ng	0.125	0.03	2	0.06	0.125	0.125	0.125	0.06	ng	0.06	0.03	n.g.
	14032	0.06	0.06	ng	ng	0.25	0.03	1	0.125	0.25	0.125	0.125	0.25	ng	0.06	0.03	0.03
	18861	0.03	0.03	ng	0.03	0.06	0.015	2	0.25	0.25	0.125	0.06	0.25	ng	0.03	0.015	0.06

	ATCC																
*S. aureus * -MSSA	29213	0.125	0.125	0.125	0.25	0.25	0.125	0.25	0.125	0.125	0.25	0.25	0.125	0.125	0.25	0.25	0.5
	18488	0.125	0.125	0.25	0.25	0.25	0.125	0.25	0.125	0.125	0.25	0.25	0.125	0.125	0.25	0.25	0.5
	18401	0.125	0.125	0.125	0.25	0.125	0.125	0.25	0.125	0.125	0.25	0.25	0.25	0.125	0.25	0.25	0.5

*S.aureus * -MRSA	18483	0.25	0.5	0.25	0.5	0.5	0.25	0.5	0.25	0.5	0.5	0.5	0.25	0.25	0.5	0.5	1
	18504	0.25	0.5	0.25	0.25	0.5	0.25	0.5	0.25	0.5	0.5	0.25	0.25	0.25	0.5	0.5	1
	18526	0.25	0.5	0.25	0.25	0.25	0.25	0.5	0.25	0.5	0.5	0.5	0.25	0.25	0.5	0.5	1

	ATCC																
*E. faecalis*	29212	0.5	0.5	1	0.5	1	0.5	1	1	1	0.5	1	0.5	0.5	1	0.5	2
	18284	1	1	2	2	2	1	2	1	2	2	2	1	1	2	2	4
	18877	1	1	0.06	0.5	2	1	2	2	2	1	2	0.5	0.5	2	0.5	4

*S. pyogenes*	17018	0.002	0.002	ng	0.002	0.002	0.002	0.004	0.002	0.002	n/t	0.004	0.004	0.004	≤0.008	≤0.008	0.015
	17019	0.004	0.004	ng	0.004	0.004	0.004	0.004	0.002	0.002	n/t	0.004	0.004	0.004	≤0.008	≤0.008	0.015
	19047	0.004	0.004	ng	0.004	0.004	0.004	0.004	0.004	0.004	n/t	0.008	0.004	0.004	≤0.008	≤0.008	0.015

	ATCC																
*S. pneumoniae*	49619	0.015	0.015	ng	0.015	0.008	0.008	0.015	0.015	0.008	n/t	0.015	0.015	0.015	≤0.008	0.015	≤0.008
	19094	0.004	0.004	ng	0.004	0.004	0.004	0.004	0.004	0.004	n/t	0.008	0.004	0.004	≤0.008	≤0.008	≤0.008
	19095	0.004	0.004	ng	0.004	0.004	0.004	0.004	0.004	0.004	n/t	0.004	0.004	0.004	≤0.008	≤0.008	≤0.008
	13345	0.125	0.125	ng	0.125	0.06	0.06	0.125	0.125	0.06	n/t	0.06	0.06	0.125	0.06	0.125	0.125
	13385	0.125	0.125	ng	0.125	0.06	0.125	0.125	0.125	0.125	n/t	0.125	0.125	0.125	0.06	0.125	0.125
	18876	0.25	0.25	ng	0.125	0.25	0.25	0.25	0.25	0.25	n/t	0.25	0.25	0.125	0.125	0.25	0.25

ATCC: American Type Culture Collection; CFUs: colony-forming units; HTM: *Haemophilus* test medium; HTMA: HTM with 1.5% agar; LHB: laked horse blood; MHA: Mueller Hinton agar; MIC: minimum inhibitory concentration; MRSA: methicillin-resistant *Staphylococcus aureus*; MSSA: methicillin-susceptible *Staphylococcus aureus*; ng: no growth; nt: not tested; REF: reference method; RMA: R.M. Alden (culture collection).

All *H. influenzae* were tested in HTM; all streptococci were tested with 2.5% LHB supplementation.
